# Molecular subgroups and biomarker guided precision immunotherapy in triple-negative breast cancer: advances, challenges, and future directions

**DOI:** 10.3389/fimmu.2026.1859601

**Published:** 2026-09-10

**Authors:** Shunhao Peng, Mengyao Qin, Gesheng Song, Qinglong Mao, Zaijie Liu, Weiwei Nie

**Affiliations:** 1 The First Affiliated Hospital of Shandong First Medical University, Jinan, China; 2 Shandong Normal University, Jinan, China

**Keywords:** biomarkers, immune checkpoint inhibitors, immunotherapy, molecular subgroups, PD-L1, precision oncology, resistance mechanisms, triple-negative breast cancer

## Abstract

Triple-negative breast cancer is an aggressive, biologically heterogeneous subtype with limited targeted therapies. Immunotherapy improves outcomes in selected patients, but durable benefit is limited by inter- and intratumoral heterogeneity, imperfect biomarkers, and primary or acquired resistance. This review integrates evidence from major positive and negative trials, molecular subgroup studies, predictive biomarkers, and tumor immune microenvironment research to assess current standards and unresolved challenges. Immune checkpoint blockade plus chemotherapy is now standard for high-risk early-stage TNBC and PD-L1-positive metastatic disease, although benefit varies by disease setting, assay, and immune phenotype. Biomarkers such as PD-L1 and tumor-infiltrating lymphocytes provide useful but incomplete guidance because of methodological differences, spatiotemporal heterogeneity, and treatment-induced changes. The central clinical challenge is to identify which patients require intensified immune-based combinations and which are unlikely to benefit from checkpoint blockade alone. We propose a precision immunotherapy framework that integrates TNBC molecular subgroups, validated and dynamic biomarker models, rational combinations, resistance mechanisms, and emerging tools, including single-cell sequencing, spatial transcriptomics, multi-omics, and AI-assisted stratification. This strategy may improve response durability while reducing unnecessary toxicity.

## 1 Introduction

Triple-negative breast cancer (TNBC) is a biologically and clinically heterogeneous subtype that lacks estrogen receptor, progesterone receptor, and human epidermal growth factor receptor 2 (HER2) expression. It accounts for approximately 15-20% of breast cancers (1). Historically, TNBC has had an aggressive course and poor prognosis because of limited actionable targets (2). Cytotoxic chemotherapy remains the treatment backbone, but its benefits are often temporary and associated with substantial toxicity, highlighting the need for more effective and durable therapies (3).

In recent years, immunotherapy, broadly defined as treatment that enhances or redirects anti-tumor immune responses, has reshaped the therapeutic landscape of TNBC. Immune checkpoints are inhibitory receptor-ligand pathways that normally restrain excessive immune activation but can be exploited by tumors to suppress T-cell function. Immune checkpoint inhibitors (ICIs), particularly antibodies targeting the programmed cell death 1 (PD-1)/programmed cell death ligand 1 (PD-L1) axis, release this inhibitory signaling and can restore anti-tumor T-cell activity. Landmark clinical trials such as IMpassion130, KEYNOTE-355, and KEYNOTE-522 have demonstrated that incorporating ICIs into chemotherapy-based regimens can improve outcomes in biomarker-defined metastatic disease and in high-risk early-stage TNBC (4, 5). These advances have established immunotherapy as a new pillar of TNBC management, while also revealing that benefit depends strongly on disease setting, biomarker selection, and the immune state of the tumor microenvironment (TME) (6–8).

Despite these advances, only a subset of patients benefit from immunotherapy. TNBC exhibits profound inter- and intra-tumoral heterogeneity. Intertumoral heterogeneity refers to biological differences between patients, whereas intratumoral heterogeneity refers to spatial and clonal diversity within a single tumor or across metastatic sites. These differences translate into highly variable immune landscapes and therapeutic responses (9). Tumor-infiltrating lymphocytes (TILs), defined as lymphocytes present within tumor nests or surrounding stroma, provide a histological readout of pre-existing anti-tumor immunity, but they vary in density, location, and functional state. Consequently, many patients experience primary resistance, while others develop acquired resistance after an initial response. Current patient selection strategies rely largely on biomarkers such as PD-L1 expression and TILs. However, their predictive performance is constrained by assay inconsistency, spatiotemporal heterogeneity, and the dynamic nature of the TME (6, 10, 11). Critical challenges also remain, including conversion of immunologically “cold” tumors into “hot” tumors and optimal management of immune-related adverse events (irAEs) (12, 13).

These limitations reveal a key gap between clinical progress and biological understanding: how can immunotherapy be tailored to TNBC complexity? Addressing this requires an integrated framework linking molecular subgroups with immune phenotypes and therapeutic vulnerabilities. Although TNBC is itself a breast cancer subtype, transcriptomic and multi-omic studies have further divided it into distinct molecular subgroups. Classification systems proposed by Lehmann, Burstein, and Fudan University identify subgroups with unique genomic and immune features (14). However, their value for immunotherapy stratification and treatment optimization remains unclear.

This review provides a comprehensive, mechanistically informed synthesis of TNBC immunotherapy. Unlike recent reviews focused mainly on ICI trials, we integrate TNBC heterogeneity, biomarker limitations, negative or discordant trial findings, resistance biology, and emerging precision-oncology technologies into a molecular subgroup-oriented therapeutic framework (**Table 1**). We first describe TNBC heterogeneity and immunogenicity, then review predictive biomarkers and approved ICIs, rational combinations, emerging immunotherapies, resistance mechanisms, toxicity and trial-design challenges, and future directions for precision immunotherapy.

**Table 1:** Novelty and scope of the present review compared with recent TNBC immunotherapy reviews.

Comparison dimension	Recent TNBC immunotherapy reviews	This review
Main focus	Usually summarize approved ICIs, major clinical trials, treatment indications, and safety profiles.	Uses molecular subgroup- and biomarker-guided precision immunotherapy as the organizing framework.
Biomarker interpretation	Mainly PD-L1, TILs, TMB, or selected single biomarkers.	Integrates PD-L1, TILs, TMB, HRD/BRCA, MSI/dMMR, immune contexture, ctDNA/MRD, and peripheral immune profiling into dynamic biomarker models.
Clinical-trial evidence	Frequently focus on positive or practice-defining trials and may discuss negative trials only briefly.	Compares positive, negative, and discordant trials, with attention to assay, disease setting, and chemotherapy backbone.
Resistance mechanisms	Often discussed as a general limitation.	Distinguishes primary and acquired resistance and links them to antigen presentation, IFN signaling, alternative checkpoints, T-cell exhaustion, immunoediting, and TME remodeling.
Spatial and single-cell technologies	Often briefly mention single-cell sequencing or spatial transcriptomics as emerging tools.	Explains how single-cell sequencing, spatial transcriptomics, and multiplex imaging can resolve immune-cell states, tumor-stromal organization, immune exclusion, and resistance evolution.
AI and digital precision oncology	AI is commonly absent or described generally as a future tool.	Discusses AI-assisted pathology, radiology, multi-omics integration, explainable AI, model transparency, external validation, regulatory and implementation barriers, federated learning, multimodal foundation models, and digital twins.

## 2 Theoretical basis and molecular landscape of immunotherapy for TNBC

TNBC is defined not only by the absence of hormone receptors and HER2 but also by a distinct immune profile that makes it a promising candidate for immunotherapy (15). Tumor mutational burden (TMB), the number of somatic mutations within a tumor, is commonly used as a surrogate for neoantigen generation. Compared with other breast cancer subtypes, TNBC generally shows higher TMB, higher infiltration levels of TILs, and more frequent PD-L1 expression (16). However, its TMB remains far lower than that of highly immunotherapy-responsive tumors such as melanoma and non-small cell lung cancer. Therefore, TMB should not be considered a stand-alone predictive biomarker in TNBC (17). Higher TIL density may indicate an anti-tumor immune response within the TME and is associated with T-cell activation (18). By contrast, high TMB and PD-L1 expression do not directly indicate T-cell activation; PD-L1 more closely reflects adaptive immune resistance and T-cell exhaustion (19). Sensitivity to immune checkpoint inhibitors is jointly determined by antigenicity, antigen presentation, T-cell infiltration, and checkpoint-mediated suppression (20, 21).

TNBC immunological diversity is also reflected in molecular subgroup classifications. The original Lehmann system identified six subtypes: Basal-like 1 (BL1), Basal-like 2 (BL2), Immunomodulatory (IM), Mesenchymal (M), Mesenchymal Stem-like (MSL), and Luminal androgen receptor (LAR). Later refinement excluded IM and MSL as tumor-intrinsic subtypes because they mainly represented immune and stromal infiltration rather than malignant-cell transcriptional programs (22). The system was therefore simplified to four subtypes (22) (**Table 2**). The Burstein classification further defined basal-like immunoactivated (BLIA) and basal-like immunosuppressed (BLIS) subtypes; BLIA shows immune activation and better prognosis, whereas BLIS shows immune suppression, reduced lymphocyte signaling, and poorer outcomes (8) (**Table 2**).

**Table 2:** Molecular subgroups, key molecular pathways, and immune-relevant characteristics of TNBC across three classification systems.

Molecular subgroup	Lehmann (2016)	Burstein (2015)	FUSCC (2019)	Key molecular pathways and features
LAR (Luminal Androgen Receptor)	LAR	LAR	LAR	High androgen receptor signaling activity; frequent mutations in PIK3CA/AKT1/mTOR pathway.
Mesenchymal	M, MSL (Mesenchymal Stem-like)	MES	MES	Enriched in cell cycle, DNA damage repair, and JAK/STAT3 signaling; expression of stem cell-related genes.
BLIS (Basal-Like Immunosuppressed)	Partially included in IM and BL2	BLIS	BLIS	High proportion of HRD; downregulation of immune-related genes, suppressed T-cell/B-cell signaling.
BLIA (Basal-Like Immunoactivated)	Partially included in IM	BLIA	–	Upregulation of IC function regulatory genes; high expression of STAT genes; high PD-L1 expression.
IM (Immunomodulatory)	IM	–	IM	Enriched in Notch signaling; PD-L1-positive (≥1%); sensitive to ICIs.
BL1/BL2 (Basal-Like 1/2)	BL1, BL2	–	–	BL1: high expression of cell cycle and DNA repair genes, often seen in BRCA mutations. BL2: overexpression of growth factor signaling and myoepithelial differentiation-related genes.
Reference	(22)	(8)	(13)	(13, 124)

The Fudan University Shanghai Cancer Center (FUSCC) classification further stratified TNBC into clinically relevant molecular subgroups, including IM, mesenchymal (MES), LAR, and BLIS groups (13) (**Table 2**). These systems differ in datasets, analytic methods, and biological emphasis, but they converge on a central principle: TNBC is not immunologically uniform. Immune-enriched subtypes such as IM or BLIA are generally associated with higher immune cell (IC) infiltration, interferon-related signaling, and antigen presentation activity, thereby providing a mechanistic basis for potentially greater sensitivity to immune checkpoint blockade (23). In contrast, BLIS tumors are characterized by immune-suppressive features (23), whereas MES or stromal enriched phenotypes may contribute to immune exclusion through extracellular matrix remodeling, fibroblast activation, and impaired T-cell penetration into tumor nests (24) (**Table 2**).

Beyond TMB, tumor immunogenicity is shaped by homologous recombination deficiency (HRD) and BRCA-associated genomic instability, which increase DNA damage and may promote neoantigen formation (25, 26); cGAS–STING activation, which induces type I interferon signaling and dendritic-cell priming (27); and interferon pathway activity, which supports antigen presentation and chemokine-mediated T-cell recruitment (28). These mechanisms may explain why some TNBCs display an ICI-sensitive “inflamed” phenotype, whereas others are “immune-excluded” or “immune-suppressed.” Understanding these associations is essential for advancing personalized TNBC immunotherapy.

## 3 Predictive biomarkers for immunotherapy in TNBC: clinical evidence, challenges, and integration strategies

### 3.1 Programmed death-ligand 1 expression

PD-1 is an inhibitory checkpoint receptor mainly expressed on activated and exhausted T cells (29), whereas PD-L1 is expressed on tumor cells and ICs in the TME, including macrophages, dendritic cells, and lymphocytes (30). The binding of PD-1 to PD-L1 suppresses T-cell effector function and promotes immune escape. PD-L1 is the most extensively validated predictive biomarker for ICI therapy in TNBC, although its clinical use remains limited by methodological and biological challenges (15). Different TNBC trials use distinct PD-L1 assays and scoring systems, affecting patient classification and cross-trial comparisons.

Two major scoring approaches have been used: IC scoring and combined positive score (CPS). IC scoring evaluates PD-L1-positive ICs in the tumor area, as in IMpassion130, where PD-L1 positivity was defined as PD-L1 expression on at least 1% of tumor-infiltrating ICs using the VENTANA SP142 assay (31). CPS incorporates PD-L1 staining on both tumor cells and ICs. It is calculated as the number of PD-L1-positive tumor cells, lymphocytes, and macrophages divided by the total number of viable tumor cells, multiplied by 100. KEYNOTE-355 used CPS, with pembrolizumab benefit defined by a CPS of at least 10 (32). These differences underscore that PD-L1 positivity is not interchangeable across studies. It depends on the antibody clone, platform, scoring system, cutoff value, and whether staining is assessed in ICs alone or in both tumor and ICs.

PD-L1 interpretation is limited by differences in assays, scoring systems, and sampling contexts (31, 33). Applying different assays to the same tumor samples yields markedly different positivity rates (31). In the IMpassion130 biomarker substudy, SP142 classified 46.4% of tumors as PD-L1-positive at IC ≥1%, whereas 22C3 and SP263 yielded higher positivity rates (9, 31). However, SP142 better predicted survival with atezolizumab, suggesting that different assays capture distinct biological features of PD-L1 expression (31). PD-L1 also shows marked spatial and temporal heterogeneity across tumor regions, metastatic sites, disease progression, and treatment (33, 34). This complexity may explain why some PD-L1-negative patients respond to immunotherapy, while some PD-L1-positive patients show primary resistance (34).

### 3.2 Tumor-infiltrating lymphocytes

In TNBC, higher stromal TIL levels consistently correlate with better prognosis and chemotherapy response (35). Clinical trials also suggest that TILs may predict immunotherapy outcomes (36, 37). In KEYNOTE-086, higher baseline TIL levels were associated with improved objective responses to pembrolizumab monotherapy (36). KEYNOTE-119 similarly showed better outcomes with pembrolizumab than chemotherapy in TIL-high patients (37). However, the association between TILs and immunotherapy response is complex and context-dependent. IMpassion130 analyses showed that TILs were most predictive in PD-L1-positive tumors, suggesting complementary rather than independent predictive value.

Clinical use of TILs as a predictive biomarker faces several challenges. No globally standardized assessment protocol exists, and methods range from visual pathological estimation to quantitative digital pathology. TILs also show marked spatial heterogeneity within tumors and may differ between primary and metastatic lesions (34). The dynamic nature of the immune response further complicates assessment, as lymphocyte infiltration may change during disease progression and after prior treatment (34, 38). Despite these limitations, TIL evaluation provides valuable biological information and may improve composite models for predicting immunotherapy response.

### 3.3 Tumor mutational burden

In TNBC, TMB should be discussed within the disease-specific context of genomic instability rather than as a generic pan-cancer biomarker. TMB quantifies the number of somatic mutations within the tumor genome and is commonly used as a surrogate for potential neoantigen generation (39, 40). TNBC is enriched for basal-like biology, HRD, TP53 alterations, and BRCA-associated genomic instability, all of which can increase mutation-associated neoantigen formation and shape immune recognition (1, 13, 40). Therefore, TMB is not a diagnostic marker of TNBC in the conventional pathological sense, but it can serve as a genomic stratification marker that helps identify TNBC subsets with greater antigenic potential.

TNBC focused genomic and multi-omic studies suggest that the value of TMB is most informative when interpreted together with molecular subgroup, DNA repair status, and immune contexture. For example, HRD/BRCA-associated tumors may have increased genomic instability, but this does not automatically translate into effective anti-tumor immunity unless antigen presentation, T-cell infiltration, and inflammatory signaling are also preserved. Conversely, a tumor with only intermediate TMB may still be immunologically responsive if it has high TIL density, PD-L1 expression, interferon-gamma (IFN-γ) signaling, and intact antigen-presentation machinery (24, 41). These observations support positioning TMB as one component of TNBC immune stratification rather than as an isolated predictor.

The clinical value of TMB in TNBC remains limited by biological and technical challenges. Most TNBCs lack the extreme hypermutation in which TMB alone strongly predicts ICI benefit, and no universal clinical cutoff has been established (42, 43). TMB estimates also vary by sequencing panel, tumor purity, bioinformatics pipeline, and cutoff definition (43). Moreover, mutation burden does not fully reflect neoantigen quality, clonality, HLA binding, expression, or antigen-presentation integrity (44, 45). Therefore, TMB in TNBC is best interpreted within a composite model integrating PD-L1, TILs, HRD/BRCA alterations, immune gene-expression signatures, antigen-presentation capacity, and dynamic markers such as circulating tumor DNA (ctDNA) (24, 41).

### 3.4 Microsatellite instability and deficient mismatch repair

MSI or dMMR status represents one of the most robust predictive biomarkers for immunotherapy response across solid tumors (46, 47). dMMR results from loss-of-function alterations in key DNA repair genes, including MLH1, MSH2, MSH6, and PMS2, leading to the accumulation of replication errors and a hypermutated phenotype, particularly within microsatellite regions (47, 48). This process can generate abundant neoantigens and create a highly immunogenic TME that is particularly susceptible to immune checkpoint blockade (48, 49).

In breast cancer, including TNBC, MSI/dMMR is rare, with an estimated prevalence of less than 2% (50). Nevertheless, MSI/dMMR remains important because it is highly actionable when present. The tissue-agnostic efficacy of pembrolizumab in MSI-H/dMMR solid tumors supports testing for this alteration in selected patients with advanced or refractory TNBC, particularly when comprehensive genomic profiling is already being performed (51). Thus, MSI/dMMR should be interpreted as a rare but clinically meaningful biomarker in TNBC. It is not useful for broad patient stratification, but it can identify a small subset of patients with a strong biological rationale for immune checkpoint blockade.

## 4 Immunotherapy modalities in TNBC: from monotherapy to combination strategies

Although immune checkpoint blockade has transformed the treatment landscape of TNBC, its benefit depends strongly on tumor immunogenicity, disease setting, biomarker status, and the therapeutic backbone used with ICIs. In unselected TNBC populations, ICI monotherapy has generally produced modest response rates, indicating that PD-1/PD-L1 blockade alone is often insufficient to generate effective anti-tumor immunity (36, 37). Therefore, most successful clinical regimens in TNBC combine ICIs with treatments that remodel the TME, increase tumor antigen release, promote dendritic-cell activation, enhance T-cell priming and trafficking, and reduce immunosuppressive barriers (34, 52).

This rationale is particularly important when interpreting the pivotal clinical trials discussed below. Chemotherapy, which remains the backbone of most approved ICI-containing regimens in TNBC, may induce immunogenic cell death (ICD), release tumor antigens and damage-associated molecular patterns (DAMPs), deplete immunosuppressive cell populations, and reduce tumor burden (52). These effects can enhance the likelihood of response to checkpoint blockade. Other therapeutic modalities, including poly ADP-ribose polymerase (PARP) inhibitors, anti-angiogenic agents, and antibody-drug conjugates (ADCs), may similarly increase tumor immunogenicity or improve IC infiltration. Thus, the clinical development of immunotherapy in TNBC should be understood not only as the testing of individual checkpoint inhibitors, but also as the rational design of immune-activating combinations.

### 4.1 Approved PD-1/PD-L1 inhibitors

In previously untreated, unresectable locally advanced or metastatic TNBC, the phase III IMpassion130 trial showed that atezolizumab plus nab-paclitaxel significantly prolonged PFS versus placebo plus nab-paclitaxel (5). In the PD-L1-positive subgroup, defined by IC PD-L1 expression ≥1% rather than tumor-cell expression, median PFS was 7.5 versus 5.0 months (HR, 0.62; P < 0.0001) (4). Overall survival (OS) also showed clinically significant differences in this subgroup, with median OS of 25.4 months and 17.9 months, respectively (53) (**Table 3**). However, later data indicated that the benefit of atezolizumab-based therapy is context-dependent.

**Table 3:** Key clinical trial characteristics and efficacy of approved or practice-defining immune checkpoint inhibitor regimens in TNBC.

Trial characteristic	IMpassion130 (Phase III)	IMpassion131 (Phase III)	KEYNOTE-355 (Phase III)	IMpassion031 (Phase III)	KEYNOTE-522 (Phase III)
Treatment Setting	First-line advanced/metastatic	First-line advanced/metastatic	First-line locally recurrent inoperable or metastatic	Neoadjuvant (early-stage TNBC)	Neoadjuvant/adjuvant (early-stage, high-risk)
Study Design	Randomized, double-blind, placebo-controlled	Randomized, double-blind, placebo-controlled	Randomized, double-blind, placebo-controlled	Randomized, double-blind, placebo-controlled	Randomized, double-blind, placebo-controlled
Patient Population	Previously untreated unresectable locally advanced or metastatic TNBC	Previously untreated unresectable locally advanced or metastatic TNBC	Previously untreated locally recurrent inoperable or metastatic TNBC	Previously untreated early-stage TNBC	High-risk stage II-III TNBC
Treatment Arms	Atezolizumab + nab-paclitaxel vs placebo + nab-paclitaxel	Atezolizumab + paclitaxel vs placebo + paclitaxel	Pembrolizumab + chemotherapy vs placebo + chemotherapy	Atezolizumab + nab-paclitaxel followed by atezolizumab + anthracycline-based chemotherapy vs placebo + chemotherapy	Pembrolizumab + chemotherapy followed by adjuvant pembrolizumab vs placebo regimen
Chemotherapy Backbone	Nab-paclitaxel	Paclitaxel	Investigator-choice chemotherapy including nab-paclitaxel, paclitaxel, or gemcitabine/carboplatin	Nab-paclitaxel followed by doxorubicin/cyclophosphamide	Paclitaxel + carboplatin followed by anthracycline/cyclophosphamide
Primary Endpoints	PFS and OS	PFS and OS	PFS and OS in CPS-defined populations	pCR	pCR and EFS
Biomarker Assessment	PD-L1 SP142, IC >=1%	PD-L1 SP142, IC >=1%	PD-L1 22C3, CPS; strongest benefit at CPS >=10	PD-L1 SP142, IC >=1%; benefit assessed regardless of PD-L1 status	PD-L1 not required for enrollment; 22C3 CPS used in analyses
Key Efficacy Results	PD-L1+: PFS 7.5 vs 5.0 mo; OS 25.4 vs 17.9 mo	Did not significantly improve PFS or OS in PD-L1+ population	CPS >=10: improved PFS and OS with pembrolizumab + chemotherapy	ITT: pCR 57.6% vs 41.1%; PD-L1+: pCR 68.8% vs 49.3%	pCR 64.8% vs 51.2%; EFS significantly improved
Treatment Benefit Pattern	Benefit concentrated in PD-L1+ disease	Negative trial; highlights backbone and context dependence	Benefit concentrated in CPS >=10 metastatic disease	Improved pCR in early-stage TNBC; benefit observed regardless of PD-L1 status and more pronounced in PD-L1-positive tumors	Benefit observed in early-stage high-risk disease regardless of PD-L1 status
Impact of the results	It provided the first positive randomized phase III evidence supporting atezolizumab plus nab-paclitaxel as first-line treatment for metastatic TNBC, particularly in PD-L1-positive disease	Explains why atezolizumab is no longer a routine metastatic standard in many settings	Defines pembrolizumab-based first-line standard for PD-L1 CPS >=10 metastatic TNBC	Supports the biological activity of atezolizumab-based neoadjuvant chemoimmunotherapy but does not define current perioperative standard practice	Defines current perioperative pembrolizumab standard
Reference	(4, 53)	(54)	(32, 58)	(57)	(5, 55)

The phase III IMpassion131 trial provided an important negative contrast: first-line atezolizumab plus paclitaxel failed to significantly improve PFS or OS versus placebo plus paclitaxel in metastatic TNBC, including PD-L1-positive disease (54). These findings explain why atezolizumab is no longer a standard metastatic TNBC option in many settings (**Table 3**). The contrasting IMpassion130 and IMpassion131 results suggest that ICI efficacy may depend on the chemotherapy backbone, steroid premedication, disease setting, PD-L1 assay and scoring, and the pre-existing immune state of the TME (31, 53, 54).

Pembrolizumab, rather than atezolizumab, currently defines perioperative immunotherapy (55, 56). In the phase III KEYNOTE-522 trial, neoadjuvant pembrolizumab plus chemotherapy followed by adjuvant pembrolizumab (53). The study met its dual primary endpoints, demonstrating statistically significant and clinically meaningful improvements in both pCR rate and event-free survival (EFS) (53). These practice-changing findings led to the approval of pembrolizumab in combination with chemotherapy as neoadjuvant treatment for patients with high-risk early-stage TNBC, followed by adjuvant pembrolizumab monotherapy after surgery (5) (**Table 3**). Similarly, the IMpassion031 trial showed that adding atezolizumab to neoadjuvant chemotherapy significantly increased pCR rates in early TNBC, irrespective of PD-L1 status (57) (**Table 4**). In metastatic disease, KEYNOTE-355 established pembrolizumab plus chemotherapy as a standard for PD-L1-positive TNBC defined by CPS >=10, significantly improving OS and PFS compared with chemotherapy alone (**Table 3**) (32, 58). These findings underscore the importance of early PD-L1 scoring and patient selection based on biomarkers.

**Table 4:** Immunotherapy and combination regimens in TNBC trials.

Study name	Treatment regimen	Patient cohort	Sample size (n)	Key efficacy outcome	Key remarks	Reference
IMpassion031	Atezolizumab + Chemotherapy	ITT	333	pCR: 57.6%	Significantly increased pCR versus placebo + chemotherapy	(57)
IMpassion031	Placebo + chemotherapy	ITT	333	pCR: 41.1%	Control arm	(57)
IMpassion031	Atezolizumab + Chemotherapy	PD-L1-positive subgroup (IC ≥1%)	152 total subgroup (77 experimental/75 control)	pCR: 68.8%	More pronounced benefit in the PD-L1-positive subgroup	(57)
IMpassion031	Placebo + chemotherapy	PD-L1-positive subgroup (IC ≥1%)	152 total subgroup (77 experimental/75 control)	pCR: 49.3%	Control arm	(57)
KEYNOTE-522	Pembrolizumab + Chemotherapy	ITT	1174	pCR: 64.8%	Established standard of care for neoadjuvant immunotherapy in early-stage, high-risk TNBC	(5, 55)
KEYNOTE-522	Placebo + Chemotherapy	ITT	1174	pCR: 51.2%	Control arm	(5, 55)
KEYNOTE-355	Pembrolizumab + chemotherapy	PD-L1 CPS >=10 metastatic TNBC	847 total trial	Improved PFS and OS versus chemotherapy alone	Practice-defining first-line metastatic regimen in CPS >=10 disease	(32, 58)
IMpassion131	Atezolizumab + paclitaxel	First-line metastatic TNBC	651	No significant PFS or OS improvement	Negative trial; supports context-dependent interpretation of atezolizumab data	(54)
IMpassion130	Atezolizumab + nab-paclitaxel vs placebo + nab-paclitaxel	Previously untreated unresectable locally advanced or metastatic TNBC	902	ITT PFS: 7.2 vs 5.5 mo; PD-L1+ PFS: 7.5 vs 5.0 mo; PD-L1+ OS: 25.4 vs 17.9 mo	Positive phase III metastatic TNBC trial; benefit most pronounced in PD-L1-positive disease	(4, 53)
MEDIOLA	Olaparib + durvalumab	Germline BRCA-mutated metastatic breast cancer, including TNBC-relevant population	34 breast cancer cohort	Objective responses and durable disease control observed	Supports PARP inhibitor + ICI rationale in DNA-repair-deficient breast cancer/TNBC context	(76)
I-SPY2 (Cohort II)	Durvalumab + Olaparib + Paclitaxel	TNBC subgroup within high-risk stage II/III HER2-negative breast cancer	372 total trial (73 experimental; 21 TNBC in experimental arm)	Estimated pCR: 47%	Exploratory combination evaluated in biomarker-enriched populations	(83)
I-SPY2 (Cohort II)	Control Chemotherapy	TNBC subgroup within high-risk stage II/III HER2-negative breast cancer	372 total trial (299 control)	Estimated pCR: 27%	Control arm	(83)
I-SPY2 (Cohort III)	Pembrolizumab + Chemotherapy	TNBC subset within high-risk ERBB2-negative breast cancer	250 total analyzed (69 experimental; 29 TNBC in experimental arm)	Estimated pCR: 60%	Validation of immunotherapy + chemotherapy efficacy	(82)
I-SPY2 (Cohort III)	Control Chemotherapy	TNBC subset within high-risk ERBB2-negative breast cancer	250 total analyzed (181 control)	Estimated pCR: 22%	Control arm	(82)
NCT00524277 (Phase II)	AE37 Vaccine + GM-CSF	TNBC subgroup (HER2 IHC 1+/2+, HR-)	25	5-year DFS: 77.7%	Exploratory subgroup signal; not statistically significant (P = 0.12)	(70)
NCT00524277 (Phase II)	GM-CSF	TNBC subgroup (HER2 IHC 1+/2+, HR-)	25	5-year DFS: 49.0%	Control arm	(70)
Camrelizumab + apatinib phase II	Camrelizumab + apatinib	Advanced TNBC	Phase II cohort	ORR: 32.5%	Supports anti-angiogenic therapy + ICI synergy in advanced TNBC	(77)
ASCENT-04/KEYNOTE-D19	Sacituzumab govitecan + pembrolizumab vs chemotherapy + pembrolizumab	Previously untreated, PD-L1-positive, locally advanced unresectable or metastatic TNBC	443 (221 vs 222)	mPFS: 11.2 vs 7.8 mo; HR 0.65; P<0.001; ORR: 60% vs 53%; median DoR: 16.5 vs 9.2 mo; OS immature	Randomized phase III trial showing significantly improved PFS with ADC-ICI therapy over chemotherapy + pembrolizumab	(90)
BEGONIA (Arm 7/8)	Dato-DXd + durvalumab	First-line unresectable locally advanced or metastatic TNBC; Arm 8 restricted to PD-L1-positive disease	95 (Arm 7 n=62; Arm 8 n=33)	ORR: 79.0% and 81.8%; mPFS: 14.0 and 11.2 mo; mDoR: 17.6 and 11.5 mo	Phase Ib/II platform study showing encouraging first-line activity with manageable safety; supports phase III development	(91)
TROPION-Breast05	Dato-DXd + durvalumab or Dato-DXd vs investigator’s choice chemotherapy + pembrolizumab	PD-L1-positive locally recurrent inoperable or metastatic TNBC	625 estimated	Ongoing; primary endpoint PFS by BICR; secondary endpoints include OS, ORR and DoR	Randomized phase III trial testing Dato-DXd-based therapy against the current pembrolizumab-chemotherapy standard	(92)
DIAMOND	Dato-DXd + durvalumab vs Dato-DXd monotherapy	PD-L1-negative advanced or metastatic TNBC	140 planned	Ongoing; primary endpoint PFS; secondary endpoints include OS, CBR, DoR, safety and quality of life	Phase II study testing whether ADC-mediated tumor killing can enhance sensitivity to PD-L1 blockade in immunologically less responsive disease	(93)

Together, these approved PD-1/PD-L1 inhibitors have established immunotherapy as a key component of TNBC treatment. However, benefits vary markedly and depend strongly on biomarker-defined populations. Some tumors achieve deep, durable responses, whereas others show primary resistance or relapse after an initial response. This clinical heterogeneity underlies immune resistance in TNBC and supports combination strategies aimed at broadening, deepening, and prolonging responses to immune checkpoint blockade.

### 4.2 Emerging immunotherapeutic modalities

Beyond approved PD-1/PD-L1 inhibitors, several novel immunotherapeutic approaches are under clinical investigation. These strategies can be divided into next-generation ICIs and non-checkpoint immunotherapies that stimulate or redirect anti-tumor immunity through other mechanisms.

#### 4.2.1 Next-generation ICIs

Beyond PD-1/PD-L1 and CTLA-4, next-generation checkpoints such as lymphocyte-activation gene 3 (LAG-3) and T cell immunoreceptor with Ig and ITIM domains (TIGIT) are promising targets for overcoming resistance (59, 60). Frequently co-expressed with PD-1 on exhausted T cells, they mediate complementary immunosuppressive pathways (59). Relatlimab, an anti-LAG-3 antibody, combined with nivolumab showed greater efficacy than nivolumab alone in advanced melanoma, leading to regulatory approval and validating LAG-3 as a therapeutic target (61). These agents, along with TIGIT inhibitors such as tiragolumab, are currently being evaluated in clinical trials for TNBC, both as monotherapy and in combination strategies (60). These strategies aim to induce durable immune activation and overcome resistance to first-generation ICIs, while ongoing studies assess biomarkers and safety (60).

#### 4.2.2 Non-checkpoint immunotherapy

Bispecific antibodies are engineered to simultaneously engage tumor-associated antigens on cancer cells and immune effector cells, most commonly CD3 on T cells, thereby redirecting cytotoxic T lymphocytes to eliminate tumors (62, 63). Although agents such as zanidatamab have advanced in HER2-positive breast cancer, their development in TNBC remains early (64). Targets of interest for TNBC include Trop-2, EGFR, and mesothelin (65). t major challenges include identifying sufficiently tumor-specific targets and limiting on-target, off-tumor toxicity (62, 65).

Therapeutic vaccines induce new or enhance existing anti-tumor immune responses (66). Vaccines targeting tumor-associated antigens such as MAGE-A and NY-ESO-1, which are commonly expressed in TNBC, have induced antigen-specific immune responses in early-phase trials, although their monotherapy efficacy remains limited (67). Neoantigen vaccines leverage tumor-specific somatic mutations and are particularly relevant in tumors with relatively high TMB, such as TNBC, offering a highly personalized therapeutic strategy (68). However, their clinical application is constrained by manufacturing complexity, cost, and treatment delays (68, 69). Peptide vaccines such as AE37, a HER2-derived peptide, have also been tested in phase II trials and may improve long-term disease-free survival in subsets of patients with HER2-low breast cancer, including TNBC (70).

Chimeric antigen receptor T-cell therapy has transformed hematologic cancer treatment but faces major barriers in solid tumors such as TNBC (71, 72). These include identifying tumor-specific antigens, achieving effective T-cell trafficking and infiltration, and overcoming the immunosuppressive TME (71, 73). Current approaches include targeting ROR1 and B7-H3, developing armored chimeric antigen receptor T cells resistant to immunosuppression, and combining them with ICIs or other therapies to improve efficacy (73, 74).

Together, these emerging approaches aim to extend anti-tumor immunity beyond conventional PD-1/PD-L1 blockade. However, their efficacy remains constrained by factors also observed in approved ICI trials, including tumor antigenicity, IC infiltration, antigen presentation, T-cell exhaustion, and local immunosuppression within the TME. Understanding why some TNBCs are immunologically responsive while others are resistant is therefore essential for designing rational combination strategies.

### 4.3 Rationale and clinical data for combination strategies

The heterogeneous clinical outcomes observed across IMpassion031, IMpassion130, IMpassion131, KEYNOTE-522, and KEYNOTE-355 indicate that TNBC is not uniformly sensitive to immune checkpoint blockade. Instead, response depends on the interaction between tumor-intrinsic features, the host immune system, and treatment-induced remodeling of the TME. After the established chemotherapy-ICI regimens discussed above, additional combinations aim to broaden or deepen response by pairing checkpoint inhibition with therapies that increase antigen release, strengthen innate immune sensing, improve T-cell trafficking, or target subtype-specific vulnerabilities (52, 75). The following subsections therefore summarize three major non-chemotherapy combination directions under active investigation in TNBC: PARP inhibitor-based combinations, anti-angiogenic combinations, and ADC-ICI combinations (76–78). These methods can be viewed, respectively, as precision therapeutic strategies targeting DNA repair defects, vascular or stroma-mediated immune exclusion, and antigen-directed delivery of cytotoxic drugs.

#### 4.3.1 Immunotherapy combined with PARP inhibitors

The rationale for combining ICIs with PARP inhibitors is strongest in HRD positive TNBC, particularly BRCA1/2 mutated tumors (79, 80). PARP inhibition causes DNA damage and cytosolic DNA accumulation, activating cGAS–STING signaling and type I interferon responses (75). This creates a more inflammatory TME with enhanced antigen presentation, T-cell infiltration, and tumor immunogenicity, thereby increasing sensitivity to ICIs (75, 81). Trials such as MEDIOLA, evaluating olaparib plus durvalumab, and I-SPY2 have shown promising activity of this strategy in TNBC (76, 82, 83) (**Table 4**).

#### 4.3.2 Immunotherapy combined with anti-angiogenic therapy

Vascular endothelial growth factor (VEGF) is a major immunosuppressive factor in the TME (84). It impairs dendritic-cell maturation, recruits immunosuppressive cells, and promotes abnormal tumor vasculature that limits T-cell infiltration (84, 85). Anti-angiogenic agents can normalize tumor vessels, improve IC trafficking, and reduce immunosuppression, thereby enhancing ICI efficacy (85, 86). In a phase II trial of camrelizumab plus the VEGFR2 inhibitor apatinib in advanced TNBC, the objective response rate was 32.5%, suggesting potential synergy (77) (**Table 4**).

#### 4.3.3 Immunotherapy combined with antibody-drug conjugates

ADCs use antigen-specific targeting to deliver potent cytotoxic payloads directly to tumor cells (87). Beyond direct cytotoxicity, some payloads, including topoisomerase I and microtubule inhibitors, induce ICD, promoting antigen release, presentation, and anti-tumor immunity (88, 89). ADCs may also modulate immune checkpoint expression, including upregulation of PD-L1, providing a biological rationale for combination with ICIs (89). Clinically, TROP-2-directed ADCs, including sacituzumab govitecan and datopotamab deruxtecan (Dato-DXd), are being evaluated with ICIs in advanced TNBC.

The ASCENT-04/KEYNOTE-D19 trial evaluated sacituzumab govitecan plus pembrolizumab versus chemotherapy plus pembrolizumab as first-line therapy for patients with previously untreated, PD-L1-positive unresectable locally advanced or metastatic TNBC. The combination significantly improved PFS compared with chemotherapy plus pembrolizumab, establishing sacituzumab govitecan plus pembrolizumab as an emerging first-line antibody–drug conjugate plus immune checkpoint inhibitor strategy in PD-L1-positive advanced TNBC (90) (**Table 4**).

Dato-DXd primarily in combination with the PD-L1 inhibitor durvalumab. In the phase Ib/II BEGONIA platform study, Dato-DXd plus durvalumab demonstrated encouraging first-line antitumor activity in unresectable locally advanced or metastatic TNBC, with high objective response rates and a manageable safety profile (91). These findings provided the rationale for the phase III TROPION-Breast05 trial, which is evaluating Dato-DXd with or without durvalumab versus investigator’s choice chemotherapy plus pembrolizumab in patients with PD-L1-positive locally recurrent inoperable or metastatic TNBC (92). In addition, the DIAMOND phase II study is comparing Dato-DXd plus durvalumab with Dato-DXd monotherapy in PD-L1-negative metastatic TNBC. This trial will test whether ADC-mediated tumor cell killing can enhance sensitivity to immune checkpoint blockade in immunologically less responsive disease (93) (**Table 4**).

## 5 Resistance mechanisms and clinical evaluation of immunotherapy for TNBC

### 5.1 Primary and acquired resistance to immune checkpoint blockade

**Figure 1:**
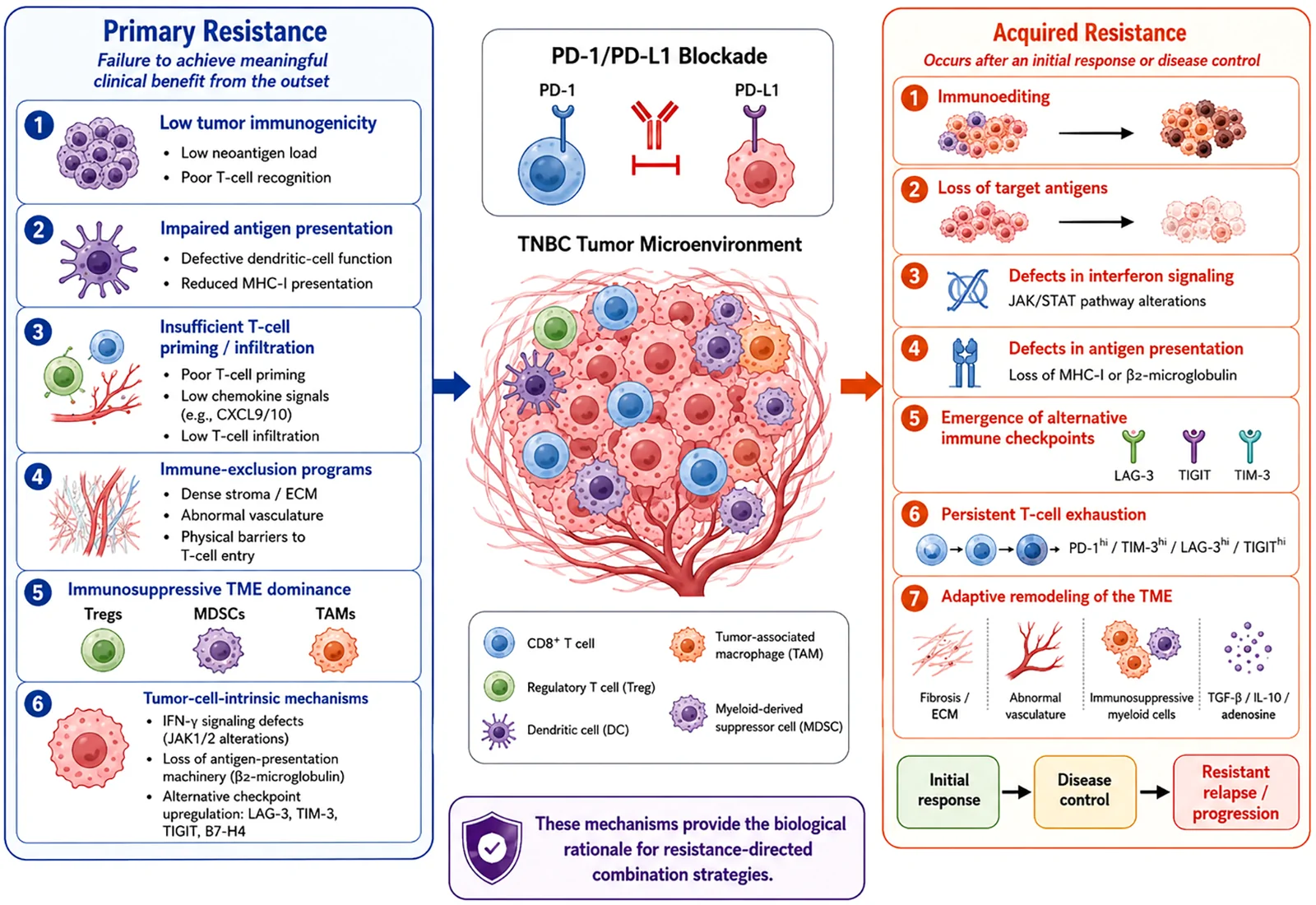
Mechanisms of primary and acquired resistance to immunotherapy in TNBC. This figure illustrates the spatial and temporal organization of immunotherapy resistance in TNBC. The central panel shows a TNBC microenvironment under PD-1/PD-L1 blockade, containing tumor cells, CD8^+^ T cells, regulatory T cells, dendritic cells, tumor-associated macrophages, myeloid-derived suppressor cells, stromal fibers, and abnormal vessels. The blue left panel summarizes mechanisms that prevent effective immune activation before response, including low immunogenicity, impaired antigen presentation, inadequate T-cell priming or infiltration, immune exclusion, an immunosuppressive TME, defective IFN-γ signaling, loss of antigen-presentation machinery, and alternative checkpoint upregulation. The orange right panel shows resistance emerging during or after an initial response, including immunoediting, antigen loss, impaired interferon signaling or antigen presentation, alternative checkpoints such as LAG-3, TIGIT, and TIM-3, persistent T-cell exhaustion, and adaptive TME remodeling. The lower bar depicts progression from initial response through disease control to resistant relapse or progression. Together, the figure integrates tumor-intrinsic, immune-mediated, and microenvironmental barriers limiting the durability of checkpoint blockade in TNBC. TNBC, Triple-negative breast cancer; PD-1, programmed cell death 1; PD-L1, programmed cell death ligand 1; TME, tumor microenvironment; IFN-γ, interferon-gamma; LAG-3, lymphocyte-activation gene 3; TIGIT, T cell immunoreceptor with Ig and ITIM domains; TIM-3, T cell immunoglobulin and mucin domain-containing protein 3.

Resistance to immunotherapy in TNBC can be broadly divided into primary and acquired resistance (94). Primary resistance refers to failure to achieve meaningful clinical benefit from the outset and often reflects low tumor immunogenicity, impaired antigen presentation, insufficient T-cell priming or infiltration, immune-exclusion programs, or dominance of regulatory T cells, myeloid-derived suppressor cells, and tumor-associated macrophages (94–96). Tumor-cell-intrinsic mechanisms, including defects in IFN-γ signaling such as JAK1/2 alterations, loss of antigen-presentation machinery such as beta-2-microglobulin, and upregulation of alternative checkpoints including LAG-3, T cell immunoglobulin and mucin domain-containing protein 3 (TIM-3), TIGIT, and B7-H4, can also render tumors intrinsically non-responsive to PD-1/PD-L1 blockade (97, 98) (**Figure 1**).

Acquired resistance develops after an initial response or disease control and reflects tumor and microenvironmental evolution under immune selection (94). Mechanisms include immunoediting, antigen loss, impaired interferon signaling or antigen presentation (97, 98), alternative checkpoints such as LAG-3 and TIGIT, persistent T-cell exhaustion, and adaptive TME remodeling (99) (**Figure 1**). These mechanisms support resistance-directed combinations, which are summarized in Section 3.3 to avoid repetition.

### 5.2 Converting “cold” tumors to “hot”: rationale and emerging strategies

A major therapeutic goal is converting immunologically “cold” TNBC into “hot” tumors. Immune-hot tumors show pre-existing anti-tumor immunity, including abundant TILs, activated cytotoxic T cells, IFN-γ signaling, intact antigen presentation, and often elevated PD-L1 expression (11, 41, 100). They are more likely to respond to ICIs because an existing immune response is restrained mainly by checkpoint pathways (11, 41). By contrast, immune-cold tumors show limited immune-cell infiltration, weak antigen presentation, low inflammatory signaling, or T-cell exclusion from the tumor bed. They respond poorly to ICI monotherapy because there is little pre-existing anti-tumor immunity to reactivate (100). The cold-to-hot transition is controlled by tumor-intrinsic and microenvironmental pathways involving antigen presentation, interferon signaling, innate immune sensing, chemokine-mediated T-cell recruitment, and stromal exclusion (99, 100). Key regulators include defective HLA class I presentation or beta-2-microglobulin, impaired IFN-JAK-STAT signaling (98), WNT/beta-catenin or PI3K/AKT activation (101, 102), TGF-beta-driven stromal exclusion (103), and checkpoints such as PD-L1, B7-H4, LAG-3, and TIM-3 (104). Together, these pathways determine whether TNBC develops a T-cell-inflamed or immune-excluded phenotype.

**Figure 2:**
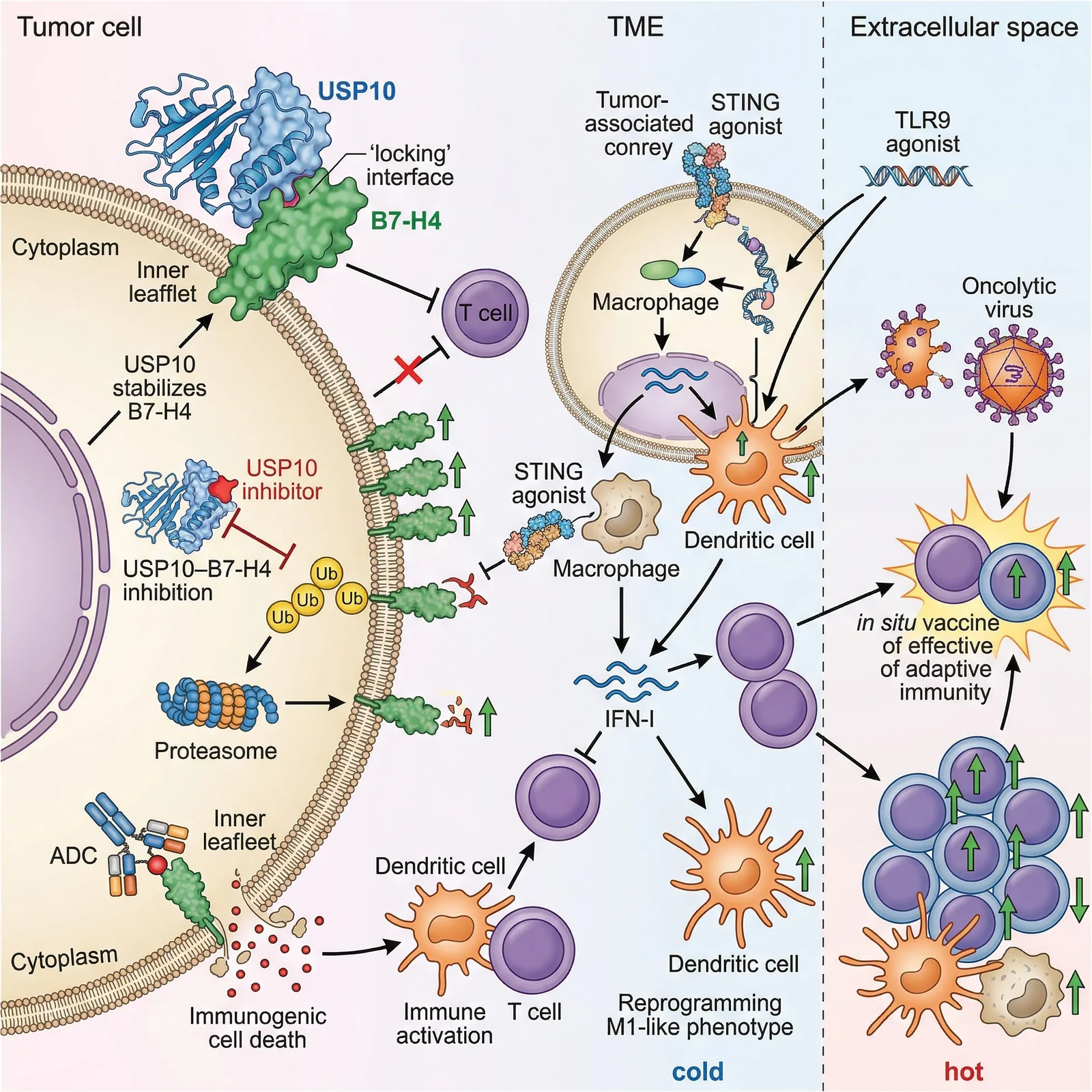
Mechanisms and strategies for converting immunologically cold tumors to hot tumors in TNBC. This process involves the USP10/B7-H4 proteolytic axis, wherein the deubiquitinase USP10 stabilizes the immunosuppressive checkpoint protein B7-H4. Pharmacological inhibition of USP10 promotes B7-H4 degradation, reactivates anti-tumor T-cell activity, and may synergize with ADCs by enhancing ICD. The diagram also illustrates local immune-reprogramming strategies. Intratumoral STING agonists activate the cGAS-STING-TBK1-IRF3 pathway, inducing type I interferon signaling, including STAT1/IRF1 activation, HLA class I and TAP1/2 upregulation, dendritic-cell maturation, and CXCL9/CXCL10/CXCL11-mediated T-cell recruitment. TLR9 agonists and oncolytic viruses can function as *in situ* vaccines by releasing tumor antigens and danger signals after tumor-cell lysis. ADCs, antibody-drug conjugates; ICD, immunogenic cell death; STING, stimulator of interferon genes; STAT1, signal transducer and activator of transcription 1; IRF1, interferon regulatory factor; HLA, human leukocyte antigens; CXCL9, C-X-C motif chemokine ligand 9; CXCL10, C-X-C motif chemokine ligand 10; CXCL11, C-X-C motif chemokine ligand 11; TLR9, Toll-like receptor 9.

A seminal study identified the USP10/B7-H4 proteolytic axis as a critical regulator of tumor immune evasion (105). The deubiquitinase USP10 stabilizes the immune checkpoint molecule B7-H4, thereby suppressing T-cell activation and promoting an immunologically “cold” TME (105). Pharmacological inhibition of USP10 promotes B7-H4 degradation, restores anti-tumor immunity, and sensitizes tumors to immune-mediated killing (105). Importantly, this axis also influences the efficacy of ADCs, as modulation of B7-H4 enhances ADC-induced ICD and promotes synergistic anti-tumor responses (88) (**Figure 2**).

Beyond single-target strategies, intratumoral/locoregional and systemic interventions are rapidly emerging (100). Intratumoral therapies are delivered directly into the tumor or tumor bed to activate local innate immune sensing. For example, Intratumoral administration of stimulator of interferon genes (STING) agonists activates the cGAS-STING-TBK1-IRF3 axis and induces type I interferon signaling within the TME (106, 107). In TNBC, relevant downstream interferon stimulated programs include signal transducer and activator of transcription 1 (STAT1)/interferon regulatory factor 1 (IRF1) activation, upregulation of antigen-presentation genes such as HLA class I and TAP1/2, and induction of T-cell-recruiting chemokines including C-X-C motif chemokine ligand 9 (CXCL9), C-X-C motif chemokine ligand 10 (CXCL10), and C-X-C motif chemokine ligand 11 (CXCL11) (28, 108). These changes promote dendritic-cell maturation, cross-presentation, CD8^+^ T-cell recruitment, and a more inflamed tumor phenotype (109, 110) (**Figure 2**).

Other intratumoral or locoregional strategies, including Toll-like receptor 9 (TLR9) agonists and oncolytic viruses, mimic pathogen-associated molecular patterns or selectively replicate in tumor cells, causing lysis, antigen release, and DAMP production (111). These approaches can induce *in situ* vaccination and systemic anti-tumor immunity, including responses in non-injected lesions (**Figure 2**). However, they differ from systemic therapies because immune activation begins locally within the treated tumor bed. In contrast, systemic strategies, including checkpoint blockade, anti-angiogenic therapy, myeloid- or stromal-targeting agents, chemotherapy, PARP inhibitors, and ADCs, can remodel immune pressure across multiple tumor sites (11, 52, 85). Thus, intratumoral/locoregional and systemic interventions are complementary but mechanistically distinct approaches for converting immunologically “cold” TNBC into “hot” tumors and overcoming primary immunotherapy resistance (34).

### 5.3 Clinical trial endpoints and evidence generation

The evaluation of immunotherapy in TNBC has necessitated a re-examination of traditional clinical trial endpoints (112, 113). In the neoadjuvant setting, pCR has been widely adopted as a primary endpoint and surrogate for long-term outcomes, as demonstrated in trials such as KEYNOTE-522 and IMpassion031 (57, 114). pCR offers the advantage of rapid readout and direct assessment of tumor eradication (114). However, its correlation with OS is not absolute, particularly in the context of immunotherapy, where delayed immune-mediated effects may influence long-term outcomes even in patients who do not achieve pCR (112).

For advanced TNBC, OS remains the gold-standard endpoint but requires large cohorts and long follow-up. Earlier-line metastatic trials such as IMpassion130 and KEYNOTE-355 often used PFS as a clinically meaningful endpoint. However, its association with OS may be influenced by subsequent therapies, crossover, delayed responses, and atypical immune-response patterns (5, 113). Alternative endpoints, including duration of response (DoR), time to treatment failure, and patient-reported outcomes, may better reflect the durability and tolerability of immunotherapy regimens (113). Future TNBC trials may also incorporate biomarker endpoints, such as ctDNA clearance, molecular residual disease, immune gene-expression signatures, and dynamic PD-L1 or TIL changes, as early indicators of efficacy and resistance (115). These approaches may enable more precise assessment of benefit in biomarker-defined TNBC populations.

### 5.4 Management of immune-related adverse events

As ICIs are increasingly integrated into both early-stage and metastatic TNBC treatment, the management of irAEs has become directly relevant to routine TNBC care. In early-stage disease, patients may receive pembrolizumab in combination with multi-agent neoadjuvant chemotherapy followed by adjuvant pembrolizumab, as in KEYNOTE-522 (55). In metastatic disease, ICIs are commonly combined with chemotherapy backbones, as in IMpassion130 and KEYNOTE-355 (32). Therefore, toxicity assessment in TNBC must distinguish immune-mediated events from chemotherapy-related adverse events such as cytopenias, neuropathy, nausea, alopecia, and hepatic enzyme elevation (116).

Common manifestations of irAEs include dermatitis, colitis, hepatitis, pneumonitis, endocrinopathies (such as thyroiditis, hypophysitis, and type 1 diabetes), and, less frequently, myocarditis or neurologic toxicities (117–119). In TNBC, endocrine irAEs are particularly important in the curative-intent setting because they may be permanent and require long-term hormone replacement even after completion of cancer therapy (55, 120). Pneumonitis and hepatitis are also clinically important because they may interrupt perioperative treatment, delay surgery, or complicate the delivery of subsequent adjuvant therapy (121).

Management requires early recognition, standardized grading using criteria such as CTCAE, and prompt intervention (122, 123). For most grade ≥2 irAEs, ICIs should be withheld and corticosteroids initiated according to severity and organ involvement (123, 124). Steroid-refractory cases may require additional immunosuppression, such as infliximab for selected colitis or mycophenolate mofetil for immune-mediated hepatitis (125). After symptom control, corticosteroids should be tapered slowly to reduce recurrence (126). In TNBC, proactive patient education and multidisciplinary coordination are essential to maintain treatment intensity when appropriate, avoid unnecessary discontinuation of potentially curative immunotherapy, and identify severe or persistent irAEs before surgery, radiotherapy, or subsequent systemic treatment.

## 6 Integrated challenges and future directions for precision immunotherapy in TNBC

### 6.1 Biomarker heterogeneity and clinical translation challenges

Immunotherapy for TNBC faces several interconnected challenges. Biomarker assessment remains limited by reliance on single biopsies and marked spatiotemporal heterogeneity (127, 128). PD-L1 expression varies with antibody clone, scoring method, cutoff, cellular compartment, metastatic site, prior therapy, and sampling time. Although TIL assessment is biologically important, globally standardized criteria are lacking, and TIL density and function may change during treatment. The neoantigen landscape reflected by TMB may also evolve clonally under immune pressure, contributing to acquired resistance (129). Spatial heterogeneity is equally substantial: different regions of the same tumor may show distinct immune infiltration and gene expression, while primary tumors, metastatic lesions, and individual metastatic sites may differ markedly (127, 130). This complexity makes systemic treatment decisions based on a single biopsy from one site and time point highly challenging. It may misclassify patients by excluding potential responders or overestimating benefit in biomarker-positive but biologically resistant tumors.

Clinical translation of biomarkers should emphasize both clinical utility and implementation feasibility. In practice, PD-L1 status, TIL density, HRD/BRCA alterations, TMB, MSI/dMMR, ctDNA/MRD dynamics, and immune profiling may help select patients for approved ICI regimens, identify candidates for PARP inhibitor, ADC, or anti-angiogenic-based combinations, support enrollment in biomarker-enriched trials, and monitor emerging resistance during treatment. However, these applications require more than biological plausibility. Analytical validation is needed to ensure reproducible specimen processing, antibody clones or sequencing panels, scoring algorithms, and quality-control procedures across laboratories (131). Clinical validation requires prospective validation in biomarker-defined TNBC cohorts rather than only in retrospective or exploratory subgroups (131). Assay standardization is particularly important for PD-L1 because IC and CPS scoring systems, antibody platforms, cellular compartments, and positivity cutoffs are not interchangeable (31); similar harmonization is required for TIL quantification, TMB estimation, HRD/BRCA testing, and ctDNA-based monitoring. Real-world use is further constrained by tissue availability, biopsy risk, turnaround time, cost-effectiveness, and unequal access to molecular diagnostics. In low- and middle-income settings, a tiered strategy may be more practical, beginning with widely available pathology-based markers such as PD-L1 and TILs and reserving multi-omics, spatial profiling, or serial ctDNA testing for situations in which the result would directly alter treatment selection or clinical-trial eligibility.

### 6.2 Limitations of current evidence

Current evidence for precision immunotherapy in TNBC remains limited by several evidence-level constraints. First, many biomarkers and biomarker-guided combinations still lack prospective validation in predefined TNBC populations, and many signals are derived from retrospective, exploratory, or subgroup analyses (132). Second, biomarker heterogeneity and assay variability remain substantial, including non-uniform PD-L1 platforms, variable TIL assessment, inconsistent TMB estimation, and limited harmonization of HRD/BRCA and ctDNA-based assays. Third, molecular subgroup classification has not yet been standardized for routine treatment selection, which limits cross-study comparison and clinical implementation. Fourth, follow-up remains insufficient for some emerging immunotherapy combinations, particularly ADC-ICI (78), PARP inhibitor-ICI (76), and novel immune-modulating strategies. Finally, cost, tissue availability, sequencing turnaround time, and unequal access to advanced diagnostics may restrict real-world applicability, especially in low- and middle-income settings (133).

### 6.3 Clinical trial designs for future TNBC immunotherapy

To translate biomarker-guided immunotherapy into clinical practice, future TNBC immunotherapy studies should increasingly use trial designs that match treatment hypotheses to molecular and immune features. Adaptive platform trials can add, drop, or prioritize treatment arms according to accumulating efficacy, safety, or biomarker signals, allowing promising combinations to be tested more efficiently, as illustrated by I-SPY2 studies in high-risk breast cancer (134). Basket trials can evaluate a shared biomarker-defined strategy, such as HRD/BRCA-directed PARP inhibitor-ICI therapy or MSI/dMMR-directed PD-1 blockade (51, 76), across tumor contexts or rare TNBC subsets. Umbrella trials can assign patients with TNBC to different immunotherapy combinations according to molecular subgroup, PD-L1 status, HRD/BRCA alteration, immune-hot or immune-cold phenotype, ADC target expression, or dominant resistance mechanism. Biomarker-enriched trials can prospectively restrict enrollment to patients most likely to benefit, improving statistical efficiency and biological interpretability. These approaches represent the future of precision immunotherapy in TNBC, but they require pre-specified assays, harmonized cutoffs, adequate tissue acquisition, clinically meaningful endpoints, longitudinal biospecimen collection, and independent validation so that exploratory biomarker signals can be translated into reliable treatment-selection strategies (135).

### 6.4 Dynamic monitoring and emerging precision-oncology technologies

**Figure 3:**
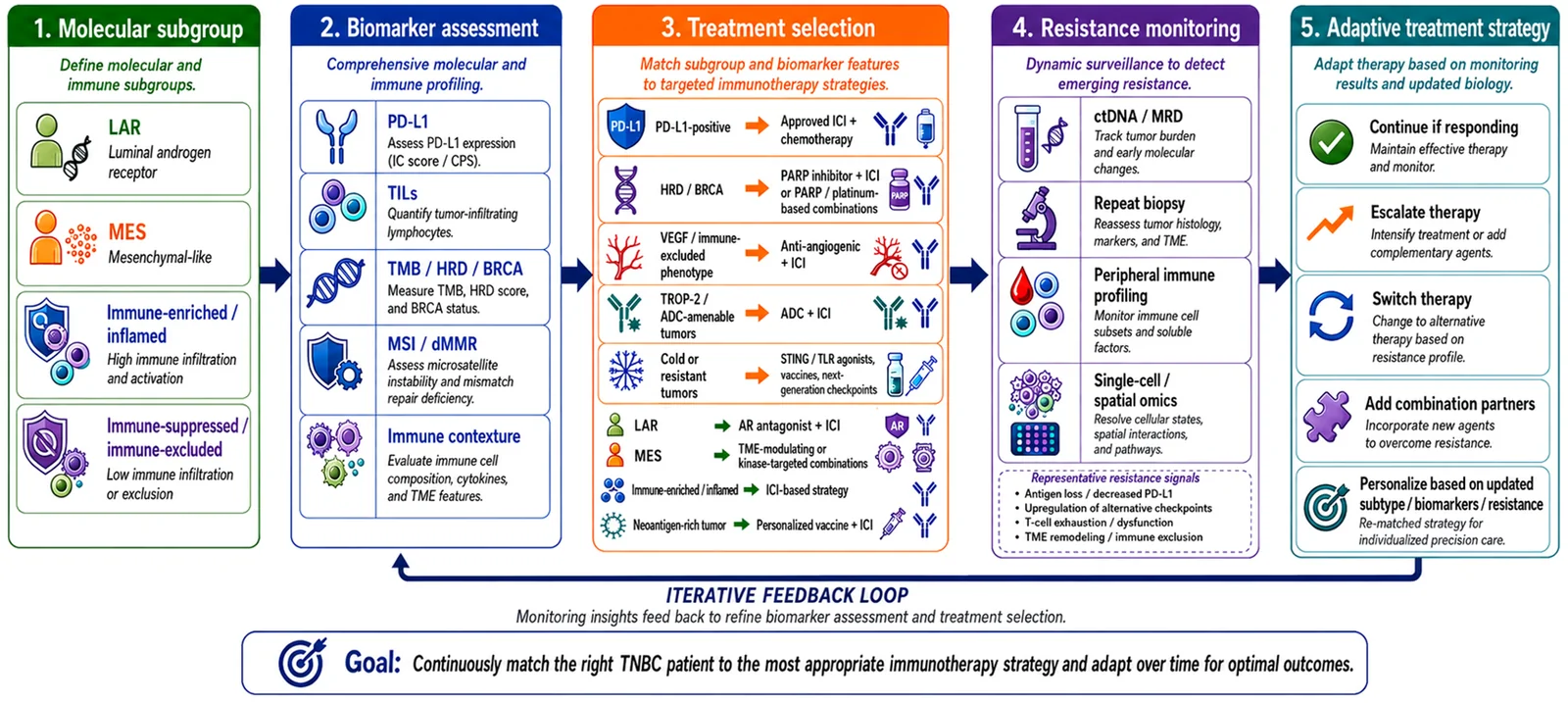
Biomarker-guided precision immunotherapy framework for TNBC. This schematic shows a five-step precision immunotherapy workflow for TNBC. Molecular subgroup and immune phenotype are first defined, followed by comprehensive biomarker assessment including PD-L1, TILs, TMB/HRD/BRCA, MSI/dMMR, and immune contexture. These results guide treatment selection, such as approved ICI plus chemotherapy, PARP inhibitor- or platinum-based combinations, anti-angiogenic therapy plus ICI, ADC plus ICI, immune-activating strategies, or subtype-directed approaches. During treatment, resistance is monitored using ctDNA/MRD, repeat biopsy, peripheral immune profiling, and single-cell/spatial omics. The updated biological information then supports adaptive strategies, including continuing, escalating, switching, or personalizing therapy. TNBC, Triple-negative breast cancer; PD-L1, programmed cell death ligand 1; TILs, Tumor-infiltrating lymphocytes; TMB, Tumor mutational burden; HRD, homologous recombination deficiency; MSI, microsatellite instability; dMMR, deficient mismatch repair; ICI, Immune checkpoint inhibitor; PARP, poly ADP-ribose polymerase; ADC, antibody-drug conjugate; ctDNA, circulating tumor DNA; MRD, minimal residual disease.

To address these limitations, future biomarker models should combine static tissue markers with dynamic monitoring (136, 137). ctDNA is a promising non-invasive tool for real-time assessment of tumor evolution (136, 138), and its clearance or emergence may predict response, relapse, and clonal evolution earlier than conventional imaging (136, 138, 139). Peripheral immune profiling, including circulating IC subsets, activation states, and soluble mediators, also enables reproducible assessment of systemic immunity and treatment interactions (140). Together, these liquid biopsy approaches support integrating tissue biomarkers, ctDNA monitoring, and systemic immune profiling into a multidimensional, real-time “tumor–immune” surveillance framework (136, 140). This strategy may shift clinical decision-making from static snapshots to dynamic longitudinal assessment, improving the precision and personalization of TNBC immunotherapy (137) (**Figure 3**).

Emerging precision oncology technologies can further strengthen this integrated approach. Single-cell RNA sequencing can distinguish tumor-cell-intrinsic programs from immune and stromal signals and identify exhausted T-cell states, suppressive myeloid populations, and rare resistant clones (141). Spatial transcriptomics and multiplex imaging can determine whether ICs are in direct contact with tumor cells or excluded to the stromal margin (142). Digital pathology and radiomics can further quantify TIL distribution, stromal architecture, necrosis, vascular patterns, and imaging phenotypes that may complement PD-L1, TMB, HRD/BRCA status, ctDNA dynamics, and immune gene-expression signatures (143, 144). Multi-omics integration can link DNA repair deficiency, copy number alteration, transcriptomic subgroup, proteomic signaling, and immune contexture (13, 24). Artificial intelligence may help integrate these heterogeneous data streams, but its clinical value depends on more than predictive accuracy. Explainable AI is needed so that model outputs can be traced to interpretable features such as immune infiltration patterns, spatial exclusion, antigen-presentation defects, radiomic signatures, or molecular alterations (145). Model transparency, calibration, external validation across institutions, prospective testing, and regulatory approval are essential before AI-based tools can guide treatment selection or trial enrollment in routine TNBC care (146, 147).

Building on this requirement for transparent and externally validated AI systems, several emerging approaches may improve the scalability and clinical relevance of precision immunotherapy models. Federated learning may enable multi-center model development without direct transfer of patient-level data, thereby supporting privacy protection, broader institutional participation, and international collaboration (148). Multimodal foundation models could then integrate pathology images, radiology, clinical records, genomics, transcriptomics, spatial data, and longitudinal treatment outcomes into unified decision-support systems for biomarker interpretation and treatment stratification (149). Digital twins represent a further extension of this concept, in which serial patient-specific data could be used to simulate tumor evolution, toxicity risk, resistance emergence, and adaptive treatment strategies (150). However, their use in TNBC immunotherapy remains investigational and will require standardized data inputs, workflow integration, health-economic evaluation, and prospective clinical validation before clinical implementation (146, 147).

### 6.5 Biomarker-driven personalized immunotherapy framework

The future of TNBC treatment depends on increasingly precise, biomarker-driven combinations. Personalized neoantigen vaccines are one promising approach. By sequencing individual tumors and identifying mutation-derived neoantigens, vaccines can be designed to elicit highly specific and durable T-cell responses (151). Given TNBC’s relatively high mutational burden, they may be used after surgery to prevent recurrence or combined with ICIs to overcome resistance (42). Personalization will also involve tailoring combinations to molecular subtypes. For example, LAR tumors may benefit from ICIs plus androgen receptor antagonists (152, 153). HRD tumors, including those with BRCA1/2 mutations, are rational candidates for combined ICI and PARP inhibition (154). MES tumors, enriched in growth factor signaling, epithelial–mesenchymal transition, and stromal remodeling, may respond to targeted kinase inhibitors or TME-modulating agents combined with immunotherapy (152, 155).

This subtype- and biomarker-driven framework moves beyond a one-size-fits-all approach. Comprehensive molecular profiling at diagnosis and progression, integrating genomic, transcriptomic, and immune data, could provide a dynamic, real-time map of tumor vulnerabilities and guide truly personalized treatment (156).

## 7 Conclusion

TNBC is an aggressive breast cancer subtype characterized by biological heterogeneity, high recurrence risk, and limited targeted therapies. Immunotherapy has reshaped its treatment landscape, with ICI-based combinations becoming standard care for high-risk early-stage disease and selected biomarker-defined metastatic settings. However, variable clinical benefit, treatment resistance, and limited biomarkers underscore the need for more precise, adaptive strategies (157). These challenges reflect the marked intrinsic and microenvironmental diversity of TNBC, which drives distinct immune phenotypes and treatment responses (24).

Future progress will require a dynamic, personalized therapeutic ecosystem. Static molecular profiling should be integrated with real-time monitoring, including ctDNA analysis, peripheral immune profiling, single-cell and spatial technologies, and AI-assisted biomarker modeling, to support adaptive treatment decisions (41, 158). Biomarker-driven trials should validate subgroup-specific combinations of ICIs with PARP inhibitors, ADCs, anti-angiogenic agents, or emerging immunomodulators. Translating next-generation approaches, including bispecific antibodies, cellular therapies, and personalized neoantigen vaccines, also remains a priority (159). As immunotherapy enters routine TNBC care, research must also address long-term safety, quality of life, treatment duration, assay standardization, cost, and equitable access. Truly individualized TNBC immunotherapy will require continuous integration of biological insights, clinical evidence, and precision technologies.
